# Protective effect of ginger (*Zingiber officinale*) against PCB-induced acute hepatotoxicity in male rats

**DOI:** 10.1039/c9ra03136g

**Published:** 2019-09-17

**Authors:** Khedher Ahd, Sabah Dhibi, Sarra Akermi, Hafsia Bouzenna, Noura Samout, Abdelfattah Elfeki, Najla Hfaiedh

**Affiliations:** Unity of Macromolecular Biochemistry and Genetics Faculty of Sciences Sidi Ahmed Zarrouk 2112 Gafsa Tunisia sabahdhibi7@gmail.com; Laboratory of Environmental Physiopathology, Valorization of Bioactive Molecules and Mathematical Modeling, Faculty of Sciences of Sfax Road Soukra km 3.5, PB no 1171-3000 Sfax Tunisia

## Abstract

After absorption by the organism, polychlorinated biphenyls (PCBs) cross cellular membranes and pass into blood vessels and the lymphatic system. It is generally in the liver, adipose tissues, brain and skin that we find the strongest concentrations of PCBs. Herbal medicine remains as a discipline intended to treat and to prevent certain functional disorders and/or pathologies caused by oxidative stress, which can be induced by pesticides, medicines or pollutants. The objective of this study is to verify the toxic and oxidative effects of PCBs and to investigate the protective effect of ginger (*Zingiber officinale*) in the liver of male rats of the “Wistar” strain. These rats are divided into 6 groups: a control group (T), two groups treated with PCB at two different concentrations (P_1_ and P_2_), a group treated with ginger extract (G), a group pretreated with ginger extract and then injected with the first concentration of PCBs (P_1_G), and a group pretreated with ginger and then injected with the second concentration of PCBs (P_2_G). The results showed that the administration of PCBs led to an increase in the relative weight of the liver, and a significant increase in all of the hepatic biomarker levels (glucose, cholesterol, triglycerides, AST, ALT, and LDH) in the serum. Furthermore, an increase in the rate of lipid peroxidation and a decrease in the antioxidant enzyme activities (catalase, superoxide dismutase and glutathione peroxidase) were observed under the influence of PCBs in the liver. The histological test showed that the PCBs induced hepatocyte vacuolization, prominent and peripheralized nuclei, hepatocellular hypertrophy and turgor of the vein in the centriacinar regions. Pretreatment with ginger extract restored all of the biochemical and oxidative parameters to the normal values and reduced the injuries caused by the PCBs. In conclusion, in our experimental conditions, ginger effectively protects the liver against the hepatotoxic effects induced by PCBs.

## Abstract 2

After absorption by the organism, polychlorinated biphenyls (PCBs) cross cellular membranes and pass into blood vessels and the lymphatic system.

## Graphical abstract

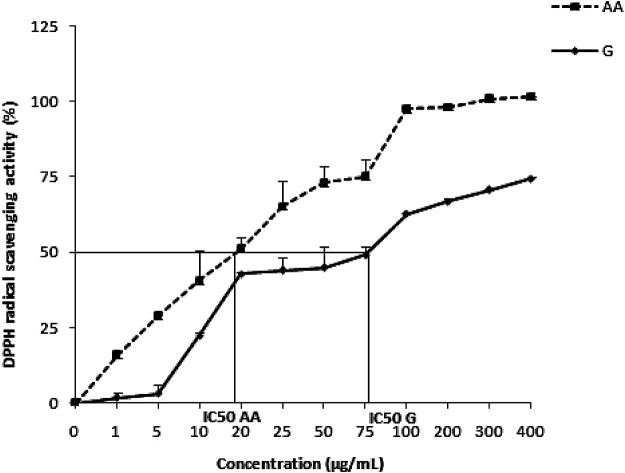

## 1. Introduction

Polychlorinated biphenyls (PCBs) are a group of POPs that include chemicals such as aldrin, heptachlor, polychlorinated dibenzo-*p*-dioxins and polychlorinated dibenzofurans, as defined by the Stockholm Convention.^1^

PCBs are a class of synthetic organic chemicals containing 209 congeners with one to ten chlorine atoms attached to the biphenyl ring, and were previously used in a variety of commercial applications.^2^ They are ubiquitous pollutants chemically characterized by their lipophilicity, high persistence, chemical stability and low biodegradability.^3^ They have a renowned toxicity; they are bioaccumulative contaminants and are found in certain fatty tissues in humans, including human milk.^4–7^

In the body, polychlorinated biphenyls cross cellular membranes and pass into the blood vessels and lymphatic system; then, they are stored in the liver, adipose tissues, brain and skin.^8,9^ PCBs can undergo anaerobic reductive dechlorination and lead to the formation of less chlorinated congeners.^10^ The highly chlorinated PCBs accumulate more in the body than the lower chlorinated ones, but they are considered less toxic.^11,12^ The biotransformation of PCBs could also occur *via* enzyme-mediated oxidation, which leads to the formation of hydroxylated polychlorinated biphenyls (OH-PCBs).^11–13^ OH-PCBs are considered to be more toxic than their parent PCBs and could lead to the disruption of thyroid hormone metabolism.^14^

Some research work has shown that PCBs as endocrine disruptors and enzyme inducers can perturb metabolism.^15^ Exposure to PCBs has harmful effects on the nervous system,^16^ reproduction because of the hormonal disruption, alteration in thyroid function, and the development of the immune system, and PCBs also have carcinogenic effects.^17,18^ Most of the past studies also reported that PCBs induce oxidative stress by the high generation of free radicals such as O_2_˙^−^ and H_2_O_2_.^19^ These ROS are thought to contribute to lipid peroxidation, DNA damage and protein degradation.^20^ Indeed, in physiological conditions, there is a perfect balance between the production of reactive oxygen species (ROS) and antioxidant defense systems. Thus, oxidative stress will be defined when there is a serious imbalance between pro-oxidants and antioxidants in favor of the latter.^21^

The liver, being the primary site for xenobiotic detoxification, is the principal target organ for toxic effects induced by environmental pollutants, including PCBs.^22^ It has been shown that polychlorinated biphenyls disrupt the function of the liver. Commercial mixtures of PCBs and their congeners have been discovered to be hepatically carcinogenic by their induction of mono-oxygenase cytochrome P450-dependent pathways in the liver.^23^ This is due to the activity of tumor initiation of the lower chlorinated congeners and tumor promoting activity of highly chlorinated PCB congeners.^24^ Furthermore, it was found that PCB_3_ is responsible for the induction of gene mutation, which is a typical characteristic of tumor initiation, in the liver and lungs of rats by increasing ROS levels.^25^

For several years, the wealth of medicinal plants was the remedy and solution to health problems and increasing attention has been paid to the protective effects of these natural antioxidants on drug-induced toxicities.

Ginger, *Zingiber officinale*, has been used for 6000 years, and it is the panacea of Asian medicine. It has been used to treat transport and pregnancy nausea, has been used as an antioxidant, and has antimicrobial and antifungal properties, in addition to its culinary uses.^26,27^ Several studies have shown that gingerol, the active ingredient of ginger, has anti-inflammatory and analgesic activities.^28^ Besides, “*in vitro*” it has been shown that *Zingiber officinale* has an antioxidant action and can protect against free radicals in animals,^29^ hence its anticancer activity.^30^ In addition, it was shown that ginger acts on the liver to reduce cholesterol biosynthesis, stimulate its conversion into bile acids and increase its fecal excretion.^31^ Therefore, we assume that ginger can prevent against the hepatotoxic effects of PCBs.

In this study, we investigated whether pretreatment with an aqueous extract of ginger for 6 weeks could prevent PCB-induced hepatotoxicity in male Wistar rats. The serum levels of glucose, cholesterol, triglycerides, lactate dehydrogenase (LDH), aspartate aminotransferase (AST) and alanine aminotransferase (ALT), and activities of antioxidant enzymes (catalase, SOD, GPx, TBARS) were measured in the liver.

## 2. Materials and methods

### 2.1. Preparation of the aqueous extract

Commercialized powder of *Zingiber officinale* (ZOE) was purchased from a local market, homogenized in boiling distilled water and soaked for about 24 h. Then the mixture was filtered, and the filtrates were stored in a refrigerator for subsequent analysis.

### 2.2. PCB sample

In this study, we used Aroclor 1260 provided from an electricity company's dead stock.

### 2.3. Phytochemical studies of *Zingiber officinale* extract

#### Determination of total phenolic content

The total phenolic content of ZOE (1 mg ml^−1^) was determined using the Folin–Ciocalteu method.^32^ Briefly, 250 μl of the extract was mixed with 0.125 ml of Folin–Ciocalteu reagent (diluted 10 times with distilled water) and 1 ml of 7.5% saturated sodium carbonate (w/v). After 2 h of incubation at 45 °C, the absorbance was measured at 765 nm by a Shimadzu 1240 model spectrophotometer. The amount of total phenolics is expressed as gallic acid equivalents (GAE, mg gallic acid per g of ZOE) through a calibration curve ranging from 0–100 μg ml^−1^ (*R*^2^ = 0.9927) and all tests were carried out in triplicate.

#### Determination of total flavonoid content

The total flavonoid content in ZOE (1 mg ml^−1^) was determined using a method described by Djeridane *et al.*^33^ A volume of 500 μl of ZOE was mixed with 150 μl of NaNO_2_ and 150 μl of AlCl_3_·6H_2_O methanolic solution (2%). Then, after 15 min of incubation at room temperature, the absorbance of the mixture was measured at 430 nm. The amount of total flavonoid content is expressed as rutin equivalents (mg RE per g of ZOE) through the calibration curve ranging from 0–400 μg ml^−1^ (*R*^2^ = 0.9644) and all tests were carried out in triplicate.

#### Determination of total condensed tannins (proanthocyanidin)

The total condensed tannins of ZOE were determined by the vanillin–H_2_SO_4_ method.^34^ 3 ml of vanillin methanolic (4%) was added to 400 μl of ZOE (1 mg ml^−1^) and 1.5 ml of concentrated sulfuric acid. The mixture was then incubated for 15 min at room temperature and the absorbance was measured at 430 nm. The amount of proanthocyanidin is expressed as catechin equivalents (mg CE per g ZOE). The calibration curve ranged from 0–350 μg ml^−1^ (*R*^2^ = 0.9978).

### 2.4. *In vitro* antioxidant activity of ZOE

#### DPPH radical scavenging activity

The method reported by Grzegorczyk *et al.*^35^ was used to estimate the free radical scavenging activity of ZOE with the DPPH radical assay. 1 ml of various concentrations of ZOE (0–400 μg ml^−1^) was mixed with 1 ml of a methanolic solution of DPPH (0.1 mM) and incubated for 30 min at 37 °C. A second range of concentrations was prepared with 1 ml of methanol to serve as a control solution. Ascorbic acid was used as a reference in the same concentration range as the test extract. Then, the absorbance of each sample was measured at 517 nm. All the analyses were done in triplicate. The ZOE antioxidant activity was calculated as follows:ARA% = 1 − [(*A*_sample_ − *A*_control_)/*A*_DPPH_] × 100where *A*_DPPH_ is the absorbance of the DPPH solution without sample extract, *A*_sample_ is the absorbance of the sample extract mixed with DPPH solution and *A*_control_ is the absorbance of the sample extract tested without DPPH. The IC_50_ value is the concentration of ZOE capable of scavenging 50% of the DPPH radicals.

#### Ferric reducing antioxidant power

The capacity of the ZOE, in different concentrations (0–500 μg ml^−1^), to reduce the ferric ion (Fe^3+^) present in the K_3_[Fe(CN)_6_] complex to a ferrous ion (Fe^2+^) was evaluated by the method described by Chu *et al.*^36^ Briefly, 2.5 ml of potassium phosphate buffer (0.1 M, pH 6) and 2.5 ml of potassium ferricyanide (1% w/v) were mixed with 1 ml of the extract (0–500 μg ml^−1^). The reaction mixture was incubated for 20 min at 50 °C in a water bath. Subsequently, 2.5 ml of trichloroacetic acid solution (10%, w/v) was added, and the mixture was centrifuged at 3000 rpm for 10 min. Then, 2.5 ml of the supernatant was mixed with 2.5 ml of distilled water and 0.5 ml of FeCl_3_ and finally incubated at 20 °C for 30 min. The absorbance of the samples was measured at 700 nm. Ascorbic acid was used as a standard for comparison and the tests were carried out in triplicate.

### 2.5. Acute toxicity test

The ZOE was investigated in toxicity studies. A total of 30 rats were divided randomly into 5 groups supplemented orally with gradually increasing concentrations (100 mg to 1000 mg per rat). The animals were directly observed for toxic symptoms, after the first 4 h of dosing. After 24 h, the surviving animals were maintained under daily observation for two weeks.

#### Live subject statement

The experimental protocol was approved by the Faculty of Ethics Committee in our institution with ethics approval number 1204. The animals were maintained in accordance with the International Guidelines for the Care and Use of Living Animals in Scientific Investigations (Council of European Communities 1986).

### 2.6. Animal treatments

Three-month-old Wistar male rats, about 111 g in body weight, fed on 15% protein food (SNA, Sfax, Tunisia), were kept in a breeding farm, at 22 °C, with a stable hygrometry, under a constant photoperiod.

These rats were divided into 6 batches each containing 6 rats:

• Group T, which served as the control group.

• Group G, which was treated by drinking 200 mg per kg b.w. of the aqueous ginger extract^37^ throughout the duration of the treatment.

• Two groups P_1_ and P_2_, which were treated with PCBs at two different concentrations (P_1_ = 470 mg per kg of b.w. and P_2_ = 980 mg per kg of b.w.) by using intra-gastric intubation,^38^ for 7 and 5 days, respectively.

• Two groups P_1_G and P_2_G, which were pretreated with the aqueous extract of ginger in drinking water for 6 weeks and then administered the PCBs at concentrations P_1_ and P_2_ for 7 and 5 days, respectively.

The animals were weighed daily and after 49 days of treatment they were sacrificed by cervical disruption. The liver was quickly removed and weighed, a portion was stored at −80 °C until analysis and a portion was fixed in formalin immediately for histopathological examination. Blood was centrifuged and serum aliquots were stored at −80 °C.

### 2.7. Preparation of the liver extracts

About 1 g of liver was cut into small pieces and homogenized in 2 ml of ice-cold Tris buffer (TBS, pH 7,4) using a crusher (homogenizing Ultra-Turax), and then centrifuged at 9000 rpm for 15 min at 4 °C. The supernatants (S1) were collected and stored at −80 °C until use.

### 2.8. Biochemical assays

#### Assays of serum markers

Serum levels of glucose, total cholesterol and triglycerides, and the activities of lactate dehydrogenase (LDH), alanine transaminase (ALT) and aspartate transaminase (AST) were assayed using commercial diagnostic kits (Spinreact Biomaghreb, Tunisia) (http://www.spinreact.com).

#### Estimation of lipid peroxidation

According to Yagi,^39^ the level of lipid peroxidation was measured as thiobarbituric acid reactive substances (TBARS). For the assay, 125 μl of supernatant (S1) was mixed with 175 μl of 20% trichloroacetic acid containing 1% butylhydroxy-toluene (BHT) and centrifuged (1000 × *g*, 10 min, 4 °C). Then, 200 μl of the supernatant (S2) was mixed with 40 μl of HCl (0.6 M) and 160 μl of thiobarbituric acid (0.72 mM) and the mixture was heated at 80 °C for 10 min.

The absorbance was measured at 530 nm. The amount of TBARS was calculated using an extinction coefficient of 156 mM^−1^ cm^−1^ and expressed in nmol mg^−1^ of protein.

#### Catalase activity

Catalase activity was measured according to Aebi.^40^ The reaction mixture (1 ml) contained 100 mM phosphate buffer (pH 7), 100 mM H_2_O_2_ and 20 μl (about 1–1.5 mg of protein) of liver homogenate. H_2_O_2_ decomposition was followed by measuring the decrease in absorbance at 240 nm for 1 min. The enzyme activity was calculated using an extinction coefficient of 0.043 mM^−1^ cm^−1^ and expressed in international units (IU), *i.e.*, in μmol H_2_O_2_ destroyed per min per mg protein, at 25 °C.

#### Superoxide-dismutase activity

The total (Cu–Zn and Mn) superoxide-dismutase (SOD) activity was determined by measuring its ability to inhibit the photo-reduction of nitroblue tetrazolium (NBT).^41^ One unit of SOD represents the amount inhibiting the photo-reduction of NBT by 50%. The activity was expressed as units per mg protein, at 25 °C.

#### Glutathione peroxidase activity (GPx)

GPx activity was assayed according to the method of Flohe & Gunzler.^42^ The activity was expressed as μmol of GSH oxidized per min per g of protein, at 25 °C.

#### Protein content

Protein content in tissue extracts was determined according to Lowry's method^43^ using bovine serum albumin as the standard.

### 2.9. Histopathological examination

Formalin-fixed livers were processed routinely, embedded in paraffin, sectioned at 3–4 μm and stained with hematoxylin and eosin (H&E). An expert in histopathological evaluation^44^ examined the slides.

### 2.10. Statistical analysis

Two independent experiments were performed. Data were expressed as means ± standard deviation (SD). Statistical significance was assessed using Student's *t*-test. *p* ≤ 0.05 was considered significant.

## 3. Results

### 3.1. Acute toxicity test

The tested animals were administered with different doses of ginger extract (100–1000 mg kg^−1^). The data of the acute toxicity test showed no toxicity or lethality observed up to 1000 mg kg^−1^. In this study, a dose of 200 mg kg^−1^ BW was chosen to investigate the antioxidant and hepatoprotective activities of the aqueous extract of *Z. officinale* in experimental animals.

### 3.2. Phytochemical studies of ZOE

The phytochemical studies of ZOE revealed a significant amount of polyphenols (179.5 ± 2.47 mg GAE per g DW ZOE) and to a lesser degree flavonoids and condensed tannins (59.1 ± 0.7 mg RE per g DW ZOE and 28.38 ± 5.66 mg CE per g DW ZOE, respectively) (Table 1).

**Table 1:** The *Zingiber officinale* extract phytochemical composition

	Total phenolics^a^ (mg GAE per g DW)	Total flavonoids^b^ (mg RE per g DW)	Total condensed tannins^c^ (mg CE per g DW)
*Zingiber officinale*	179.5 ± 2.47	59.1 ± 0.7	28.38 ± 5.66

a Total phenolic content as the gallic acid equivalent.

b Total flavonoid content as the rutin equivalent.

c Condensed tannin as the catechin equivalent. Values are expressed as mean ± standard deviation (*n* = 3).

### 3.3. *In vitro* antioxidant capacity

#### DPPH radical scavenging activity

**Fig. 1:**
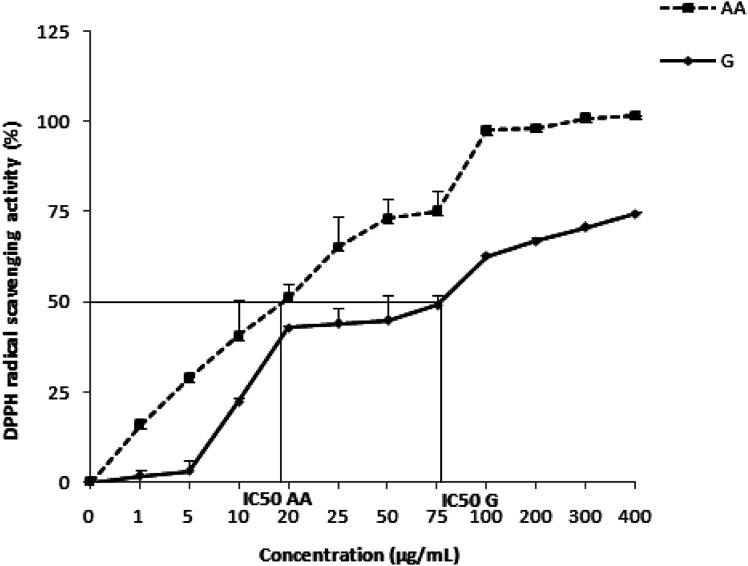
The antiradical activity of *Zingiber officinale* against the radical DPPH. Values are represented as mean ± standard deviation (*n* = 3).

The antiradical activity of the *Zingiber officinale* extract, *in vitro*, against the DPPH radical is shown on Fig. 1. Indeed, as the concentration of the extract increases, the anti-DPPH activity increases also until reaching a maximum concentration of 0.4 mg mL^−1^. The antioxidant capacity was determined from IC_50_, which corresponds to the concentration necessary to reduce 50% of the DPPH radicals. The IC_50_ of the *Z. officinale* extract is mathematically calculated and valued as 79.70 ± 0.27 μg mL^−1^, which is significantly lower than that of ascorbic acid used as a positive control (19.41 ± 2.71 μg mL^−1^) (Table 2).

**Table 2:** The *Zingiber officinale* extract antioxidant capacity^a^

	DPPH (IC_50_, μg mL^−1^)	FRAP (EC_50_, mg mL^−1^)
*Zingiber officinale*	79.70 ± 0.27	19.4 ± 1.23
Ascorbic acid	19.41 ± 2.71	0.47 ± 0.002

a Values are expressed as mean ± standard deviation (*n* = 3).

#### Ferric reducing antioxidant power

**Fig. 2:**
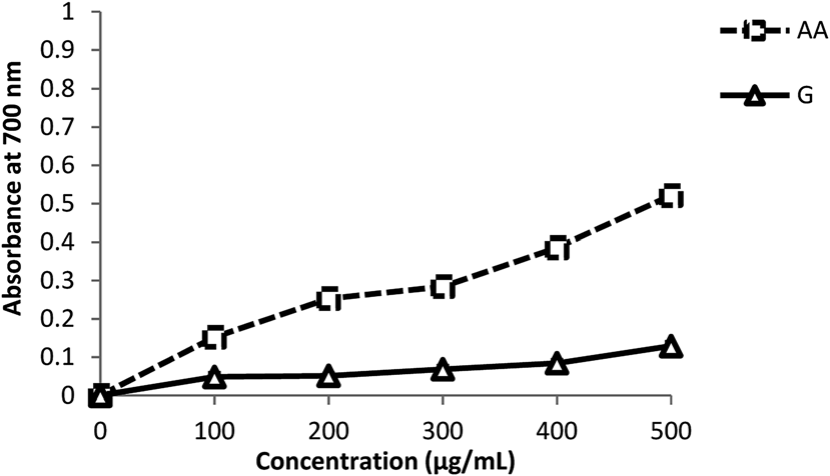
The reducing power of *Zingiber officinale* and ascorbic acid by the FRAP assay. Values are expressed as mean ± standard deviation (*n* = 3).

As shown in Fig. 2, ginger has the capacity to reduce Fe^3+^ to Fe^2+^, at different concentration ranges. The reducing power of ZOE and its concentration are dose-dependent. It was found to be 0.129 ± 0.008 absorbance units at 500 μg mL^−1^ with the effective concentration EC_50_ being 19.4 ± 1.23 μg mL^−1^. This activity appeared significantly (*p* < 0.05) lower compared with that of the positive control (ascorbic acid), which was 0.52 ± 0.003 absorbance units at 500 μg mL^−1^ with an EC_50_ of 0.47 ± 0.002 μg mL^−1^.

### 3.4. Liver weight

**Fig. 3:**
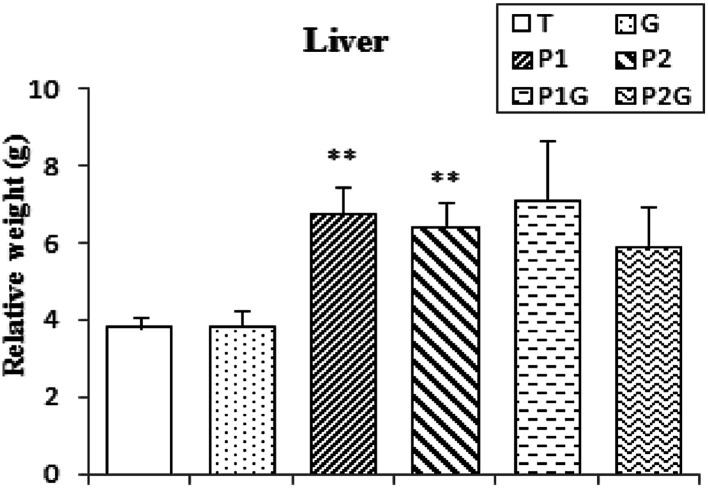
Liver relative weight of control rats (T), rats consuming ginger (G), rats treated for 7 and 5 days (P_1_ and P_2_, respectively) and rats pretreated with ginger for 6 weeks (P_1_G and P_2_G). Values correspond to the mean of 6 measurements ± SD. Student test: **(*p* ≤ 0.01) indicates significant differences between (P_1_ and P_2_) and control rats (T).

After sacrifice of the rats, the livers were weighed and the relative weight was estimated. The results showed an important increase in liver relative weight in all PCB-treated groups: P_1_, P_2_, P_1_G and P_2_G (+77%, 68%, 87% and 54%, respectively). There was no significant improvement noticed even with ginger pretreatment (Fig. 3).

### 3.5. Serum markers of cell damage

**Fig. 4:**
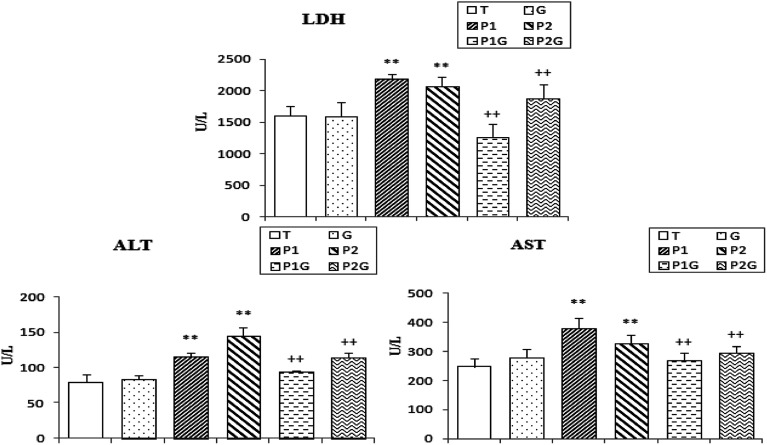
Activities of lactate dehydrogenase (LDH), alanine aminotransferase (ALT) and aspartate aminotransferase (AST) in control rats (T), rats consuming ginger (G), rats treated for 7 and 5 days (P_1_ and P_2_, respectively) and rats pretreated with ginger for 6 weeks (P_1_G and P_2_G). Values correspond to the mean of 6 measurements ± SD. Student test: **(*p* ≤ 0.01) indicates significant differences between (P_1_ and P_2_) and control rats (T). ^++^(*p* ≤ 0.01) indicates significant difference between (P_1_G and P_2_G) and (P_1_ and P_2_) rats.

Lactate dehydrogenase (LDH), aspartate aminotransferase (AST) and alanine aminotransferase (ALT) are released into the blood when certain organs or tissues, particularly the liver and heart, are injured. As shown in Fig. 4, PCB treatment induced a significant increase in the serum levels of LDH, AST and ALT (+33%, 42% and 64%, respectively) in (P_1_) and (P_2_) rats, as compared to controls (T). These effects were significantly decreased in the PCB-treated rats drinking ginger extract (P_1_G and P_2_G groups) (−26%, 20% and 19%, respectively) compared to the PCB-treated groups (Fig. 4).

The serum levels of glucose (+24%), triglycerides (+180%) and total cholesterol (+98%) were also found to be significantly increased in (P_1_) and (P_2_) rats, as compared to controls. However, these levels were significantly decreased in the rats pretreated with ginger extract (P_1_G and P_2_G) (Table 3).

**Table 3:** Serum levels of glucose, triglycerides and cholesterol in control rats (T), rats consuming ginger (G), rats treated for 7 and 5 days (P_1_ and P_2_, respectively) and rats pretreated with ginger for 6 weeks (P_1_G and P_2_G)^a^

	Controls (T)	G	P_1_	P_2_	P_1_G	P_2_G
Glucose (mmol L^−1^)	4.8 ± 0.98	5.36 ± 0.86	6.84 ± 0.46**	5.04 ± 0.05	5.58 ± 0.73^+^	5.1 ± 0.14
			+42.5%	+5%	−18%	
Triglycerides (mmol L^−1^)	0.65 ± 0.12	0.48 ± 0.16	2.18 ± 0.2**	1.69 ± 0.44**	1.07 ± 0.73^++^	1.11 ± 0.12^++^
			+200%	+160%	−51%	−34%
Total cholesterol (mmol L^−1^)	1.56 ± 0.14	1.82 ± 0.08	3.04 ± 0.53**	3.14 ± 0.24**	2.19 ± 0.73^++^	1.88 ± 0.11^++^
			+95%	+101%	−28%	−40%

a Student test: **(*p* ≤ 0.01) indicates significant differences between (P_1_ and P_2_) and control rats (T). ^++^(*p* ≤ 0.01) indicates significant difference between (P_1_G and P_2_G) and (P_1_ and P_2_) rats. Values correspond to the mean of 6 measurements ± SD.

### 3.6. Estimation of lipid peroxidation levels (TBARS) in liver extract

**Fig. 5:**
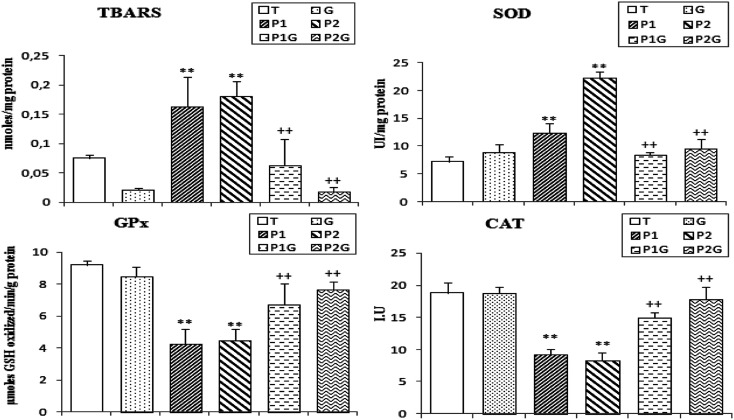
Levels of TBARS and the activities of superoxide-dismutase (SOD), glutathione peroxidase (GPx) and catalase (CAT) in the liver of control rats (T), rats consuming ginger (G), rats treated for 7 and 5 days (P_1_ and P_2_, respectively) and rats pretreated with ginger for 6 weeks (P_1_G and P_2_G). Values correspond to the mean of 6 measurements ± SD. Student test: **(*p* ≤ 0.01) indicates significant differences between (P_1_ and P_2_) and control rats (T). ^++^(*p* ≤ 0.01) indicates significant difference between (P_1_G and P_2_G) and (P_1_ and P_2_) rats.

In this study, we showed that the administration of PCBs (at concentrations P_1_ and P_2_) induces an important increase in the TBARS rates (+117% and 140%, respectively) in the liver as compared to control rats (T). However, in rats pretreated with singer extract (P_1_G and P_2_G), the rates of TBARS decreased and the results are comparable with those obtained for the control rats (T) (Fig. 5).

### 3.7. Changes of antioxidant enzyme activities in liver extracts

PCB treatment induced a significant increase (+142%) in SOD activity in the liver of (P_1_) and (P_2_) rats and about a −53% decrease in CAT and GPx activities (Fig. 5). However, the ginger extract pretreatment (P_1_G and P_2_G groups) showed important changes in the activities of these enzymes (−45% decrease in SOD activity, +88.5% and 63.5% increase, respectively, in CAT and GPx activities).

### 3.8. Liver histopathological changes

**Fig. 6:**
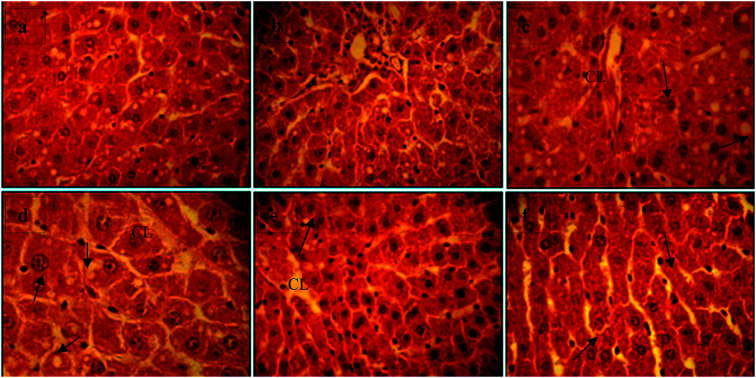
Liver histopathological changes induced by exposure to PCBs. Representative photographs showing (a) a control rat (T) liver section, (b) a liver section from a rat consuming ginger (G), (c) and (d) liver sections from PCB-treated rats (P_1_ and P_2_) and (e) and (f) liver sections from ginger-pretreated rats (P_1_G and P_2_G). H&E staining; (a), (b) and (d)–(f) 40× objective; (c) 100× objective. CL: turgescence of the centrilobular vein. Arrows: vacuolization of hepatocytes, prominent and peripheralized nuclei and hypertrophy and turgor of the central vein.

Histological examination showed that the PCBs (for both concentrations of 470 mg kg^−1^ and 940 mg kg^−1^) lead to an increase in the size of the hepatic lobules due to hepatocyte hypertrophy characterized by large areas of cytoplasmic pallor (hydropic degeneration), mild vacuolation of hepatocytes (arrows, c and d, Fig. 6), prominent and peripheralized nuclei, and hypertrophy and turgor in the central vein (CL, c and d, Fig. 6). These changes were moderately improved in ginger-pretreated rats (e and f). In the control (a) and ginger groups (b), there was no evidence of hepatic abnormality; we observed mild cytoplasmic clearing without vacuolization and with centrally located nuclei consistent with glycogen.

## 4. Discussion

In this study, we intended to determine the toxic effect of polychlorinated biphenyls (PCBs) on hepatic function, and to verify the protective effects of ginger extract against the toxicity induced by PCBs.

In the animal study, we found that the oral administration of PCBs to male rats from the “Wistar” strain induced liver hypertrophy. In accordance with our study, Lai *et al.*^45^ explained this liver hypertrophy by the accumulation of lipids due to disruption of hepatic lipid intake and metabolism.

The PCB treatment induced a highly significant increase in the serum levels of glucose, cholesterol and triglycerides, which is associated with an increase in the hepatic biomarkers AST, ALT and LDH. This could be explained by the strong accumulation of PCBs in the liver and the severe alteration of hepatocytes. Indeed, the transaminases (AST and ALT), which are involved in protein renewal and the synthesis of new peptides, strongly affect the metabolism due to their inhibition by PCBs. Consistent with our results, Pereira & Rao^46^ found that the administration of PCBs (Clophen A60) at 2.8 mg per kg of b.w. per day significantly increased the level of glucose, LDH, cholesterol and triglycerides. In addition, our results are harmonized with other studies reporting negative effects of PCBs in the liver, such as that by Wang *et al.*^47^ AST, being a primarily mitochondrial enzyme, allows us to deduce that, at the cellular level, there was an increase in respiratory burst and mitochondrial involvement in the hepatocytes of rats treated with PCB. Pereira & Rao^46^ explained the increase of hepatic biomarkers with the increase of lipid peroxidation that could affect mitochondrial function and the leakage of mitochondrial enzymes due to the injury of the mitochondrial membranes.

After 49 days of treatment, we observed in rats pretreated with ginger aqueous extract (P_1_G and P_2_G) that the toxicity of PCB was greatly reduced. The aqueous extract of ginger decreased significantly the AST, ALT, cholesterol, triglyceride, glucose and LDH levels. Several studies clearly reinforce our results, showing the hepatoprotective effect of ginger against liver toxicity induced by ethanol, carbon tetrachloride, bromobenzene and acetaminophen, accompanied by a significant decrease of AST and ALT.^48–51^ It is the same for the hypocholesterolemic effect of ginger, which is probably due to the inhibition of cellular synthesis of cholesterol. The possible mechanism of the plant to reduce serum triglycerides is due to the increase in the expression and activity of the lipoprotein lipase enzyme in the vessels. This enzyme increases the breakdown of triglycerides in the blood vessels and reduces the blood levels of triglycerides.^52^ Ginger also inhibits hepatic fatty acid and triglyceride synthesis by lowering key enzyme activity.^53^ In the research of Heeba & Abd-Elghany,^54^ it was also shown that ginger stimulates the conversion of cholesterol into bile acids, and increasing the excretion of cholesterol and phospholipids in the stools after taking ginger can also be considered as a potential mechanism for the effects of ginger in reducing serum cholesterol levels.^55,56^ In the study of Gao *et al.*,^57^ it was shown that ginger improves insulin sensitivity in the body, which may explain the mechanism of the decrease in blood glucose levels in rats pretreated with ginger.

Our experimental study showed that the induction of oxidative stress by PCBs has been demonstrated by the highly significant increase of TBARS in liver tissues. We found that the levels of lipid peroxidation are responsible for the formation of lipid hydroperoxides in membranes, leading to lesions in the membrane structure and inactivation of enzyme membrane binding.^58^ Research by Twaroski *et al.*^59^ showed that the toxic manifestations induced by PCBs may be associated with the high production of ROS and the initiation and self-propagating reaction of lipid peroxidation. The oxygen radicals react with poly-unsaturated fatty acid residues in the phospholipids resulting in the production of excessive amounts of products, which can damage proteins and DNA. In fact, PCBs may interact with hydrogen peroxide to form hydroxyl radicals, which are the most active form of ROS in biological systems.

It seems quite clear that the presence of ginger reduced the TBARS in pretreated rats (P_1_G and P_2_G). This urged us to deduce that ginger plays a protective role against oxidative stress induced by PCBs. The research of Rajkumar & Rao^60^ showed an important inhibition of lipid peroxidation by dehydro-zingerone, which is a synthetic analogue of zingerone. It is important to note that dietary ginger concomitant to 1% w/w during the administration of malathion (20 ppm) for 4 weeks significantly attenuated lipid peroxidation in the liver.^61^

The high lipid peroxidation may also be due to decreased activities of catalase and superoxide dismutase, which are scavenger enzymes of free radicals. In our study, we found that PCBs increased the activity of superoxide dismutase and decreased the catalase and glutathione peroxidase activities. Several studies have associated changes in the activity of superoxide dismutase with the reduced synthesis, high degradation or inactivation of this enzyme.^16,20,62^ Other studies have investigated the increase in superoxide dismutase activity and they explained these results by the increase of the concentration of superoxide anions (O_2_˙^−^), which causes an increase in the concentration of H_2_O_2_ due to the inhibition of catalase and glutathione peroxidase, as they are the responsible enzymes for scavenging H_2_O_2_. In the work of Venkataraman *et al.*,^16^ the decrease in glutathione peroxidase activity in rats treated with PCBs is correlated to the reduction of the substrate, meaning reduced glutathione (GSH) level and high peroxide level. In other research studies, the decrease observed in our results is evaluated by decreased synthesis and/or inactivation of the enzyme.^20^

However, the administration of ginger as a pretreatment (200 mg per kg of b.w.) for 6 weeks restored the activities of antioxidant enzymes. This could be explained by the presence of several antioxidant compounds in ginger, such as gingerols, shogaol derivatives, ketones, phenolics, flavonoids and volatile oils.^58^ [6]-Gingerol as the major constituent of ginger has been shown to exert an inhibitory effect on xanthine oxidase, the enzyme responsible for the generation of reactive oxygen species.^63^ This antioxidant activity of ginger extract is explained by the results found in our phytochemical study, which revealed significant levels of polyphenols and a lesser amount of flavonoids and tannins, which is correlated with the findings of Gabr *et al.*^64^ These antioxidant substances are well known to protect the body against free radicals. The antioxidant activity of *Z. officinale* aqueous extract was evaluated *in vitro* using the DPPH and FRAP tests. Our results showed important reducing properties due to the presence of compounds that reduce the ferricyanide complex of Fe^3+^ to the ferrous (Fe^2+^) form by donating a proton. In addition, the ginger aqueous extract exhibits a significant scavenging activity based on the reduction of the stable and free radical DPPH (purple color) thanks to the hydrogen atoms existing on the antioxidant contents of the ZOE. Our observations are consistent with those demonstrated by Gabr *et al.*^64^ These antioxidant potentials of the ginger extract might be due to its richness in polyphenols and flavonoids. These bioactive compounds are known by their redox properties and might play an important role in chelating transition metals and scavenging free radicals.^65^ The studies of Shanmugam *et al.*^58^ showed that the activities of antioxidant enzymes SOD and GPx were improved in the liver tissue of animals pretreated with an ethanolic extract of ginger at a concentration of 100 mg per kg b.w. compared to animals exposed to bromobenzene. The same research team reported that the extract of ginger decreases the activity of cytochrome P450 by reducing the metabolism of bromobenzene into reactive metabolites.

Histological examination showed that the PCBs (at both concentrations of 470 mg kg^−1^ and 940 mg kg^−1^) induced vacuolization of hepatocytes, prominent and peripheralized nuclei and hypertrophy and turgor of the central vein. The results of this study are consistent with the works of Lai *et al.*^45^ and Wang *et al.*,^47^ which showed severe changes in hepatocytes with an increase in the size of the lobules and capsular irregularity. However, the aqueous extract of ginger slightly attenuated these cytological manifestations. This has been demonstrated in the studies of Heeba & Abd-Elghany,^54^ showing that the administration of ginger plays an important role in reducing liver damage and preserves the integrity of the hepatocyte membranes.

In conclusion, it seems that ginger is able to protect the liver against oxidative stress and biochemical manifestations induced by polychlorinated biphenyls. The high protection capacity of ginger could be due to its content of antioxidant phytochemicals, which neutralize the high production of free radicals generated by the PCBs.

## Data availability

The data used to support the findings of this study are available from the corresponding author upon request.

## Conflicts of interest

The authors declare that there are no conflicts of interest.

## Supplementary Material
